# Synergies between mycorrhizal fungi and soil microbial communities increase plant nitrogen acquisition

**DOI:** 10.1038/s42003-019-0481-8

**Published:** 2019-06-21

**Authors:** Rachel Hestrin, Edith C. Hammer, Carsten W. Mueller, Johannes Lehmann

**Affiliations:** 1 Soil and Crop Sciences, School of Integrative Plant Science, Cornell University, Ithaca, NY 14853 USA; 2 Department of Biology, Lund University, Box 118, 22100 Lund, Sweden; 3 Lehrstuhl für Bodenkunde, TU München, 85356 Freising-Weihenstephan, Germany; 4 Atkinson Center for a Sustainable Future, Cornell University, Ithaca, NY 14853 USA; 5 Institute for Advanced Studies, TU München, 85748 Garching, Germany

**Keywords:** Soil microbiology, Arbuscular mycorrhiza, Element cycles

## Abstract

Nitrogen availability often restricts primary productivity in terrestrial ecosystems. Arbuscular mycorrhizal fungi are ubiquitous symbionts of terrestrial plants and can improve plant nitrogen acquisition, but have a limited ability to access organic nitrogen. Although other soil biota mineralize organic nitrogen into bioavailable forms, they may simultaneously compete for nitrogen, with unknown consequences for plant nutrition. Here, we show that synergies between the mycorrhizal fungus *Rhizophagus irregularis* and soil microbial communities have a highly non-additive effect on nitrogen acquisition by the model grass *Brachypodium distachyon*. These multipartite microbial synergies result in a doubling of the nitrogen that mycorrhizal plants acquire from organic matter and a tenfold increase in nitrogen acquisition compared to non-mycorrhizal plants grown in the absence of soil microbial communities. This previously unquantified multipartite relationship may contribute to more than 70 Tg of annually assimilated plant nitrogen, thereby playing a critical role in global nutrient cycling and ecosystem function.

## Abstract (web summary)

Rachel Hestrin et al. examine the synergies between mycorrhizal fungi and soil microbes that mediate plant nitrogen acquisition. They show that non-additive effects of fungi and soil microbes may be responsible for more than half of the nitrogen that arbuscular mycorrhizal plants derive from organic matter.

## Introduction

Nitrogen (N) is a limiting nutrient in many natural and managed ecosystems^1,2^. Arbuscular mycorrhizal (AM) fungi form symbioses with the majority of terrestrial plants and can substantially enhance plant N acquisition from soil, thereby potentially alleviating plant N limitation and playing an important role in plant productivity and soil nutrient cycling^3–9^. Although they appear to lack the genetic machinery necessary for decomposition, AM fungi can acquire a substantial quantity of mineral N from organic matter^6–14^. A growing body of literature implicates other soil biota with decomposer capabilities as key players in AM fungal N acquisition and transfer to plants^15^. However, it is not clear whether multipartite AM-microbial interactions result in competitive versus synergistic nutrient acquisition^11,14,16,17^ and how these interactions respond to global environmental changes such as N enrichment.

Terrestrial ecosystems experience substantial N enrichment due to atmospheric deposition and fertilizer applications, with consequences for soil organic matter dynamics, microbial biodiversity, plant community composition, and primary productivity^16–21^. Long-term N enrichment of grassland soils results in substantial changes in microbial community structure and functional gene representation^17–20^. Although it is recognized that these changes may have important implications for ecosystem function, the particular mechanisms through which long-term N enrichment influences plant-biotic interactions and plant productivity are not fully understood. In order to account for these relationships in Earth system models and predict ecosystem response to increasing N enrichment, it is necessary to understand the extent to which AM-microbial interactions mediate plant N acquisition and associated biogeochemical processes^22–25^.

Here we show that multipartite synergies between AM fungi and soil microbial communities substantially enhance plant and fungal N acquisition from organic matter and microbial acquisition of plant photosynthates. Long-term N enrichment disrupts these synergies, resulting in diminished mycorrhizal N acquisition from organic matter. These results have implications for terrestrial nutrient cycling models, agricultural management, and our understanding of ecosystem response to global change.

## Results

### Plant N acquisition from organic matter

**Fig. 1:**
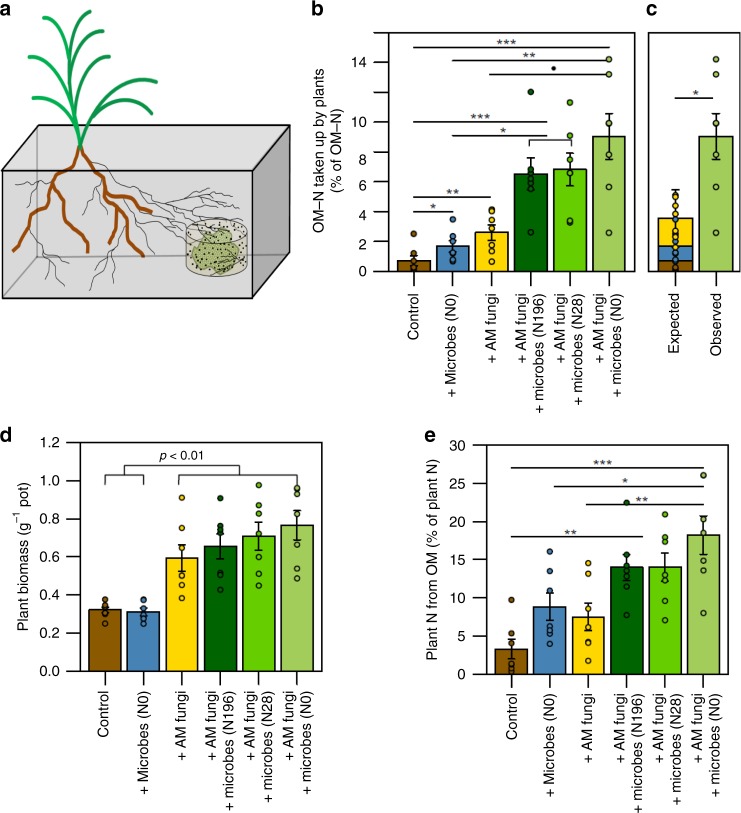
Multipartite synergies between AM fungi and soil microbial communities increase plant biomass and N acquisition from organic matter. **a** Mesocosm design. **b** Plants acquired more N from organic matter in the presence of AM fungi and soil microbial communities. **c** Plants grown with both AM fungi and soil microbes acquired more N than expected based on the sum of N acquired by control plants and those grown with AM fungi or soil microbes alone. **d** AM colonization is associated with greater plant biomass. **e** AM plants grown with soil microbes derived a greater proportion of their total N from organic matter than control plants and plants grown with AM fungi or soil microbial communities alone. Significance levels are indicated with the following symbols: ·*p* < 0.1, *p < 0.05, **p < 0.01, ***p < 0.001 and denote the results of a Tukey’s HSD test performed on log-transformed data (**b**, **d**), an unpaired *t* test (**c**), and a Tukey’s HSD test performed on untransformed data (**e**). Error bars represent the standard error (*n* = 7 biologically independent samples)

We used stable isotopes, plant mesocosms, AM fungi, and soil microbial communities collected from an N gradient experiment to investigate how multipartite interactions influence plant N acquisition from organic matter and how these relationships respond to long-term N enrichment. *Brachypodium distachyon* seeds were planted in double-autoclaved sand and gravel with or without spores of the AM fungus *Rhizophagus irregularis* (formerly *Glomus intraradices*). After one month, the root systems of plants that had been inoculated with spores were colonized by the fungus. AM and non-AM plants were then transplanted into mesocosms containing a double-autoclaved sand-gravel mixture and a patch of ^15^N/^13^C-enriched organic matter (Fig. 1a). An inoculum of fresh grassland soil containing whole soil microbial communities that had been exposed to an N enrichment gradient for eight years (annual N additions of 0, 28, or 196 kg N ha^−1^; Kellogg Biological Station Long-Term Ecological Research Site, Hickory Corners, MI) was added to the organic matter in a subset of the mesocosms.

The 6 mesocosm treatments included: plants that were grown without any additional AM fungi or soil inocula [control], plants grown only with microbial communities from an unfertilized field [ + microbes (N0)], plants grown only with AM fungi [ + AM fungi], plants grown with AM fungi and microbial communities from a field fertilized with 196 kg N ha^−1^ per year [ + AM fungi + microbes (N196)], plants grown with AM fungi and microbial communities from a field fertilized with 28 kg N ha^−1^ per year [ + AM fungi + microbes (N28)], and plants grown with AM fungi and microbial communities from an unfertilized field [ + AM fungi + microbes (N0)]. These 6 treatments were each replicated 7 times. To control for abiotic soil characteristics, double-autoclaved soil from the unfertilized field was added to the AM and non-AM mesocosms that did not receive a microbial inoculum of fresh soil. Weekly addition of a low-N-modified Hoagland’s solution minimized competition for non-N nutrients between plants and microbes and provided sufficient N to keep the plants alive for a duration of time similar to a natural growing season. A total of 15.75 mg of inorganic N was added to each mesocosm over the course of the experiment. This experimental design allowed us to assess the individual and combined contributions of AM fungi and the rest of the soil microbial community to plant N acquisition from organic matter, and investigate the legacy of environmental N enrichment on plant-biotic interactions and nutrient acquisition strategies.

As expected, multipartite relationships between plants, AM fungi, and free-living soil microbes were associated with greater plant N acquisition from organic matter. Surprisingly, the synergies emerging from these interactions far exceeded an additive effect on plant N acquisition. While plants grown with either soil microbes or AM fungi acquired twofold and threefold more N from the organic matter than control plants, respectively, plants grown with both soil microbes and AM fungi acquired ten to twelvefold more N from the organic matter than control plants (Fig. 1b). This ten to twelvefold increase in plant N acquisition is more than double the expected increase in plant N acquisition based on the sum of N taken up by plants grown with free-living soil microbes or AM fungi alone (Fig. 1c). This synergistic effect on plant N acquisition represents an emergent property of plant-biotic relationships and underscores the extent to which complex, multipartite interactions can influence mycorrhizal ecology and nutrient acquisition.

### AM-microbial mediation of organic matter cycling

By modifying plant nutrient acquisition, these multipartite plant-biotic interactions also play an important role in terrestrial organic matter cycling and soil carbon (C) storage. In the presence of both free-living soil microbial communities and AM fungi, plants derived up to 18% of their total N from organic matter—double the proportion of plant N derived from organic matter when plants grew with free-living soil microbes or AM fungi alone, even after differences in total plant biomass were accounted for (Fig. 1d, e). This demonstrates that AM-microbial interactions may shift plant N acquisition strategies, resulting in greater relative acquisition from organic versus mineral nutrient stocks. Although plant-biotic synergies that enhance mineralization and uptake of N from organic sources may result in the loss of some soil organic matter, it is also possible that they simultaneously stimulate greater primary productivity and associated soil organic matter formation through root and mycorrhizal C inputs^26^. The net effect of AM-microbial interactions on soil C may be an important component of the recently observed relationship between mycorrhizal type and soil C storage and warrants further research^9,12,21^.

### Plant and AM fungal N acquisition pathways

**Fig. 2:**
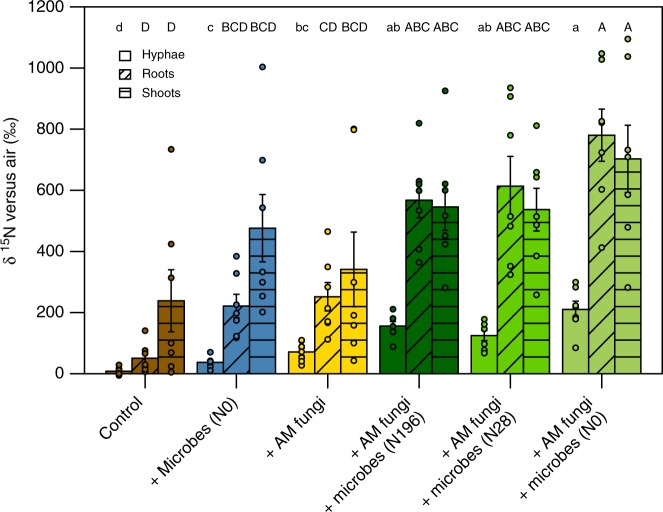
Relative ^15^N enrichment of fungal hyphae, plant roots, and plant aboveground tissue. Lowercase letters denote the results of a Tukey’s HSD test comparing log-transformed mean δ ^15^N values of fungal hyphae; uppercase letters denote the results of a Tukey’s HSD test comparing mean δ ^15^N values of plant tissues (*p* < 0.05). Error bars represent the standard error (*n* = 7 biologically independent samples)

Greater plant N acquisition may have occurred through direct root uptake or, for AM plants, through a combination of direct root uptake and AM hyphal uptake and transfer to plants (see Supplementary Notes for further discussion on plant N uptake)^27,28^. The root systems of plants grown with free-living soil microbes, AM fungi, and both free-living microbes and AM fungi were successively larger than those grown without, thereby providing a more extensive root network with which the plants could take up mineralized nutrients. Mycorrhizal plants acquired a greater proportion of N from organic matter compared to the AM fungi themselves, further suggesting that increased N acquisition by AM plants was largely due to direct N uptake through their extensive root systems rather than through an exchange with AM fungi (Fig. 2). However, it is also possible that AM fungi acquired and transferred N from organic matter to plants without incorporating the same proportion of N into their own biomass. Although potentially disadvantageous to fungal growth in a N-limited environment, this fits within the context of the mycorrhizal symbiosis, wherein AM plants reward their fungal partners for mineral nutrients with photosynthates^29^.

Our observation that AM plants took up proportionally more N from the organic matter than did the AM fungi themselves provides a useful insight into plant and mycorrhizal nutrient acquisition strategies under conditions when the nutrient source is physically accessible to both the plants and fungi. Many other studies focused on AM contributions to plant N acquisition from organic matter utilize experimental designs that exclude roots from the N source. While these studies provide important mechanistic insight into mycorrhizal relationships, multipartite interactions (both competitive and synergistic) may differ when all organisms and nutrient sources are present together rather than isolated from each other. Although AM fungi can deliver N to their plant hosts, our results also provide evidence for a synergistic feedback through which mycorrhizally-driven plant growth promotion increases direct root uptake of N, thereby providing resources for additional photosynthate production, which can then be used to stimulate further mycorrhizal or microbial activity that supports continued plant growth and rhizodeposition.

### Synergies between AM fungi and free-living microorganisms

**Fig. 3:**
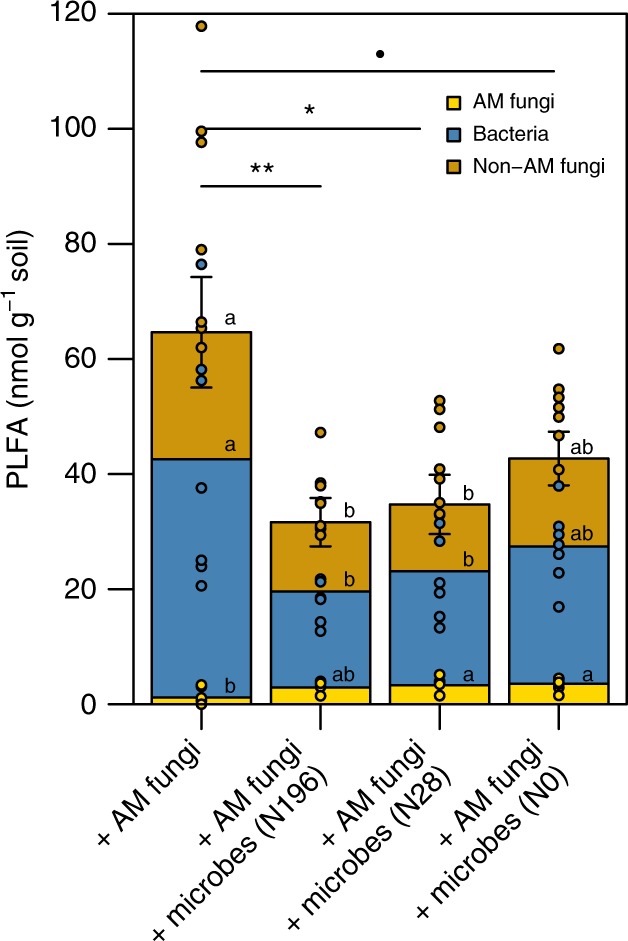
Microbial lipid biomass present in organic matter. Phospholipid fatty acid (PLFA) analysis was used to measure microbial lipid biomass in the organic matter harvested from mesocosms containing AM plants only and both AM plants and free-living soil microbes from grasslands fertilized with 0, 28, and 196 kg N ha^−1^ per year. Significant differences between total microbial lipid biomass measured through a Tukey’s HSD test performed on log-transformed PLFA sums from each treatment are indicated by the following symbols: ·*p* < 0.1, *p < 0.05, **p < 0.01. Error bars represent the standard error of the mean of total microbial PLFAs measured in each mesocosm type (*n* = 7 biologically independent samples). Microbial lipid biomass associated with AM fungi, bacteria, and non-AM fungi is indicated in yellow, blue, and orange bars, respectively. Lowercase letters above the upper right-hand corner of each bar denote the results of Tukey’s HSD tests performed only for PLFAs of the same subtype (AM fungi, bacteria, or non-AM fungi; *p* < 0.05). N enrichment did not result in a substantial difference in the ratio of fungal:bacterial lipids present in mesocosms containing AM plants and microbial inoculum from grassland fields. However, the ratios of AM fungal:bacterial lipids in these mesocosms were higher than in mesocosms inoculated only with AM fungi, suggesting that the presence of soil microbial communities benefitted the AM fungi in addition to benefitting the plant. It is not clear whether this was a direct benefit to the AM fungi, or whether it was modulated through increased provision of plant photosynthates

**Fig. 4:**
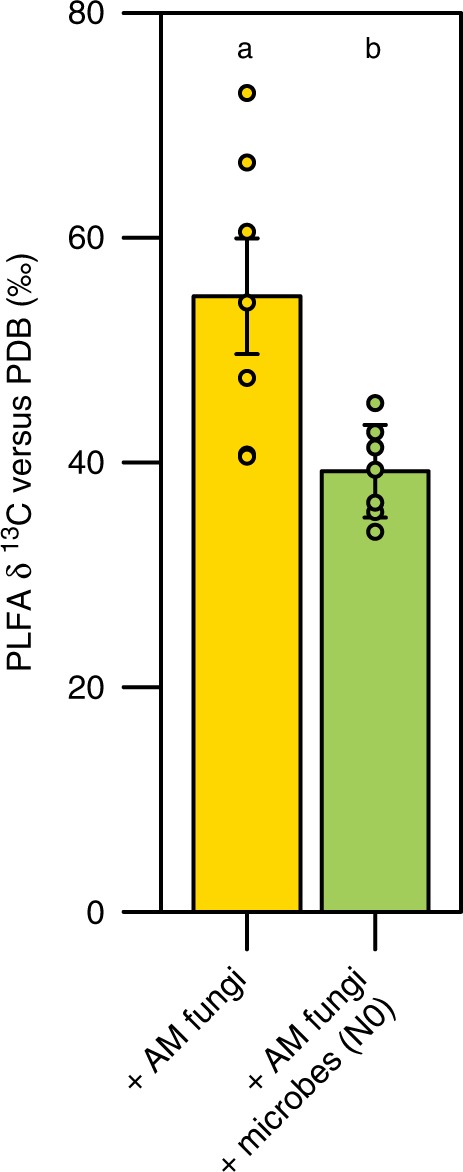
Mean relative ^13^C enrichment of microbial biomass lipids measured through phospholipid fatty acid (PLFA) analysis. Since organic matter was enriched with ^13^C and plant photosynthates were depleted in ^13^C, lower PLFA δ ^13^C values suggest that microbes derived a greater proportion of their C from plant photosynthates. Letters denote the results of a Tukey’s HSD test performed on log-transformed data; error bars represent the standard error (*p* < 0.01, n = 7 biologically independent samples)

Multipartite interactions between plants, AM fungi, and free-living soil microbial communities had a synergistic effect on plant and AM fungal productivity and N acquisition. Other studies have shown that AM fungi and free-living soil biota can inhibit one another and do not consistently enhance plant N acquisition from organic matter when plants are denied direct access to the organic matter^15,30^. However, in our work, where plants were permitted access to organic matter along with AM fungi and free-living soil microbial communities, AM fungal lipid biomass was greater in organic matter harvested from mesocosms containing both AM plants and free-living soil microbial communities than from mesocosms without free-living soil microbes (Fig. 3). Furthermore, both plants and AM fungi acquired more N from organic matter in the presence of free-living soil microbes (Figs. 1 and 2). This suggests that the free-living soil microbial community had a synergistic rather than an inhibitory effect on both AM plants and the AM fungi themselves. Depleted ^13^C values in the microbial lipid biomass and fungal hyphae extracted from mesocosms containing both AM plants and soil microbial communities indicate that both AM fungi and free-living soil microbes derived a greater proportion of their C from plant photosynthates when all players were present (Fig. 4 and Supplementary Table 1)^31^. From this, we can infer that the free-living soil microbial community also benefitted from this multipartite plant-biotic relationship, under which enhanced rhizosphere C inputs were available for microbial utilization belowground. Additional research is necessary in order to evaluate the net effect of AM-microbial synergies and associated plant N acquisition on soil C stocks.

**Fig. 5:**
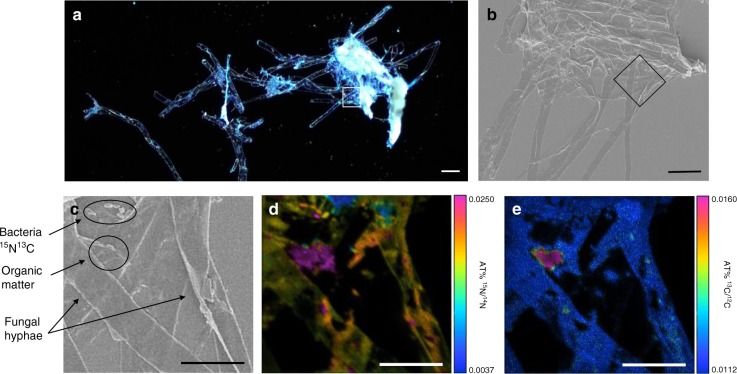
Light microscopy, scanning electron microscopy (SEM), and nano-scale secondary ion mass spectrometry (NanoSIMS) images of enriched organic matter, AM fungi, and soil microbes. **a** Light microscopy image of fungi and soil microbes grown in ^15^N^13^C enriched organic matter. The white square demarcates the 30 × 30 µm region from which the NanoSIMS images were collected. **b** SEM image of the same sample. The black square demarcates the same 30 × 30 µm region from which the NanoSIMS images were collected. The dense cluster towards the top of the image is organic matter. The 5-10 µm thick strands extending below are fungal hyphae. **c** SEM image of the exact region from which NanoSIMS images were collected. A cluster of bacterial cells is located in the top left corner. The 5–10 µm thick strands extending across the image are fungal hyphae. ^15^N^13^C enriched organic matter is located in the upper left quadrant of the image. **d** NanoSIMS images of ^12^C^15^N/^12^C^14^N and (**e**) ^13^C/^12^C isotope ratios of fungi, bacteria, and organic matter are shown in a color scale with natural abundance values in blue (.003676 and .0111802, respectively) and high enrichment in purple. Bacterial ^15^N incorporation was highly heterogeneous between cells, even within the distance of a few microns. Fungal ^15^N incorporation was relatively even across hyphae. Scale bars, 30 µm (**a**, **b**) and 10 µm (**c**–**e**)

Nano‐scale secondary ion mass spectrometry (NanoSIMS) provided direct evidence of the route through which multipartite synergies drive soil organic matter decomposition and biotic N acquisition. NanoSIMS images collected of microbes growing in the organic matter at the end of the experiment showed the spatial distribution of ^15^N and ^13^C within the soil microbial community (Fig. 5 and Supplementary Fig. 1). While both fungi and bacteria were enriched with ^15^N, bacterial cells were more enriched in ^15^N than neighboring fungal hyphae. This suggests that both fungi and bacteria acquired N from the organic matter, but that compared to fungi, bacteria derived a greater proportion of their N from the organic matter. This provides direct evidence of the intermediary role that bacteria play in mineralization of organic matter into those N species that may later be available for uptake by plants and fungi lacking saprotrophic capabilities. The relative depletion of ^15^N in fungal hyphae compared to bacterial cells growing near ^15^N-enriched organic matter also illustrates that fungal hyphal networks may access nutrients across broader spatial scales than individual bacterial cells. The isotopic N ratio of fungal hyphae shown in Fig. 5 likely represents an integration of different N sources taken up by hyphae located farther away from the ^15^N-enriched organic matter than the bacterial cells nearby.

### N enrichment disrupts multipartite synergies

A legacy of environmental change due to N enrichment disrupted the multipartite synergies contributing to plant N acquisition from organic matter. Compared to AM plants grown with soil microbial communities from unfertilized fields, AM plants grown with microbial communities that had developed under N fertilization were smaller and acquired less N from organic matter (Fig. 1b-e and Fig. 2). Since our mesocosms provided an environment with equal nutrient content in all treatments, this demonstrates that the effects of N enrichment on microbial function and microbially-mediated plant nutrient acquisition persist even once the primary disturbance is no longer evident (i.e., once mineral N is no longer abundant). Other authors have found that N enrichment of grassland soils is associated with lower microbial biomass, a reduction in the relative abundance of AM fungi and oligotrophic bacteria, an increase in the relative abundance of archaea and copiotropic bacteria, and concomitant changes in microbial N cycling^16–20^. In some cases, these changes have been decoupled from soil N concentrations measured at the time of sampling, providing further evidence that N enrichment has a lasting effect on microbial function^19,20^. The inhibitory effect that we observed of long-term N enrichment on microbially-mediated plant N acquisition supports these findings and demonstrates that this legacy effect has implications for plant-biotic synergies and ecosystem primary productivity.

**Fig. 6:**
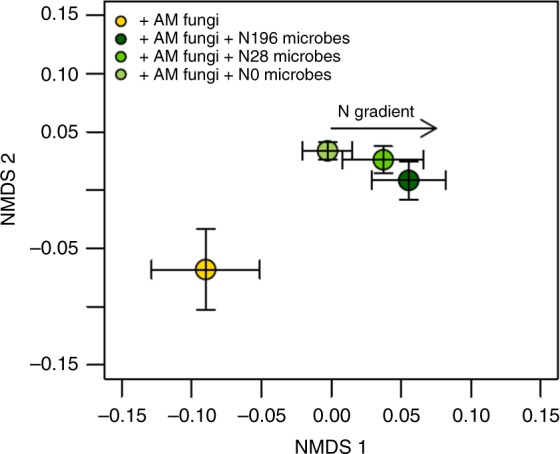
Nonmetric multidimensional scaling (NMDS) plot of microbial community composition based on PLFA profiles. Microbial community composition in mesocosms containing AM plants only (yellow symbol) and those containing AM plants and soil microbes that developed under an environmental N gradient of 0, 28, and 196 kg N ha^−1^ per year (light, medium, and dark green symbols, respectively) varied significantly (*p* < 0.01). Error bars show the standard error of the mean NMDS scores (*n* = 7 biologically independent samples)

We expected to find that a lasting inhibitory effect of N enrichment on microbially-mediated plant N acquisition from organic matter would be associated with N-driven decreases in microbial biomass and decomposition activity^12,13,18,19,32,33^. However, total microbial lipid biomass, as measured by phospholipid fatty acid (PLFA) analysis, was not consistently associated with greater plant N acquisition from organic matter (Figs. 1b and 3). The composition of microbial lipid biomass was significantly different across treatments (Fig. 6, p < 0.01). This suggests that although total microbial lipid biomass was not correlated with plant N acquisition, differences in abundance of particular microbial groups may have been responsible for the synergistic interactions leading to enhanced plant N acquisition.

**Fig. 7:**
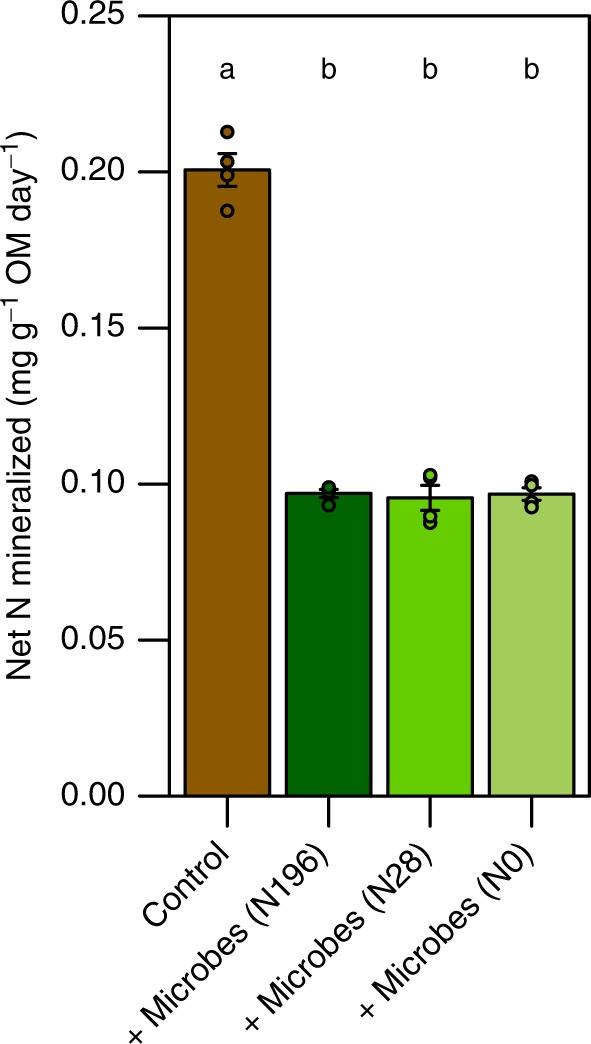
Microbial net N mineralization potential. In the absence of plants and AM fungi, net N mineralization rates did not vary between microbial inocula sampled from switchgrass fields fertilized with 0, 28, and 196 kg N ha^−1^ per year. Lower N mineralized in treatments containing microbial inocula compared to controls suggests that some of the N mineralized was immobilized by microbes. Statistical significance is based on a Tukey’s HSD test (*p* < 0.001); error bars represent the standard error (*n* = 4 biologically independent samples)

We expected that differences in the composition of microbial communities that developed under N enrichment would be associated with differences in microbial N mineralization capacity. When assessed in the absence of plants and AM fungi, there was no difference between the net N mineralization capacity of soil microbial communities that had developed under varying N enrichment (Fig. 7). However, N enrichment history was associated with differences in microbially-mediated plant N acquisition from organic matter (Fig. 1). This suggests that the microbial processes leading to enhanced plant N acquisition are sensitive to N enrichment but that the ecological and biogeochemical impacts of this environmental change emerge more strongly in the presence of plants. It is not clear whether this plant-dependent response is due to differences in plant provision of photosynthates to symbiotic and free-living soil microbes or other mechanisms. Given the widespread history and predicted future of global N deposition, these results have important implications for terrestrial N cycling and ecosystem function^34^. They also suggest that N fertilization history may have a long-lasting inhibitory effect on plant access to organic N sources, with potential consequences for agricultural productivity.

## Discussion

Our results demonstrate that emergent synergies between plants, mycorrhizal fungi, and free-living soil microbes have a highly non-additive effect on plant N acquisition from organic matter. Although these relationships have been explored in previous studies, the role of the synergies between AM fungi and free-living microorganisms in plant N acquisition and soil organic matter cycling has not been quantified directly^3,6^. Here we show that more than half of the N that AM plants derive from organic matter may be attributed to a synergistic relationship between AM plants and soil microbial communities and that this synergy is disrupted by a history of N enrichment. Applied to estimates of global plant N uptake, these results suggest that more than 70 Tg of annually assimilated plant N can be attributed to interactions between AM plants and soil microbes, but that these relationships are sensitive to environmental change^35^. These findings can be used to constrain Earth system models and improve agricultural management, where organic inputs provide an important supply of N to plants. Since terrestrial ecosystems are often N-limited, this also has implications for global N cycling and net primary productivity^36,37^.

## Methods

### Plant and mycorrhizal establishment

*Brachypodium distachyon* seeds were surface sterilized with ethanol and planted in cones filled with 1:1 mixtures of double-autoclaved sand and gravel (v:v) at near-neutral pH. For AM fungal inoculation, 500 spores of the AM fungus *Rhizophagus irregularis* (previously *Glomus intraradices*) were added at the time of seeding^38^.

### Mesocosms and experimental design

After ~1 month, plants were transplanted from cones into mesocosms containing a double-autoclaved mixture of sand and gravel. One gram of ryegrass leaves containing 0.37 mg ^15^N and 5.06 mg ^13^C were mixed with double-autoclaved sand and buried in an open container at one end of the mesocosm. For treatments with soil microbial inocula, 0.25 g of fresh soil from perennial switchgrass (*Panicum virgatum* L.) fields that had been fertilized with three different levels of N (0, 28, and 196 kg N ha^−1^ per year; Kellogg Biological Station Long-Term Ecological Research Site, Hickory Corners, MI) for eight years was added directly to the organic matter^20^. For treatments without live soil microbial inocula, 0.25 g of double-autoclaved soil was added to the organic matter to control for any potential effect of abiotic soil components. Mesocosms were watered carefully to limit direct transport of N solutes from the organic matter. Each treatment was replicated seven times; replicates were arranged in a spatially distributed randomized block design. Regular addition of a low-N-modified Hoagland’s solution reduced competition for non-N nutrients between plants and microbes (0.5 mM KCl, 0.5 mM CaCl_2_, 0.5 mM Ca(NO_3_)_2_, 0.5 mM KNO_3_, 1 mM MgSO_4_, 50 µM NaFe EDTA, 20 µM KH_3_PO_4_, 10 µM H_3_BO_3_, 0.2 µM Na_2_MoO_4_, 1 µM ZnSO_4_, 2 µM MnCl_2_, 0.5 µM CuSO_4_, 0.2 µM CoCl_2_, 25 µM HCl, and 0.5 mM MES buffer).

Surface-sterilized seeds and autoclaved growing medium were used in order to minimize the background microbial community present in the mesocosms^13,14,24^. Additionally, the mesocosms were watered only with reverse osmosis filtered water and kept in a growth chamber inside a limited-access plant growth facility. However, the growing conditions were not completely sterile and some microbes may have been introduced into the mesocosms over the course of the experiment. Although fresh grassland soils likely contained some AM fungal spores or hyphae, the roots of plants harvested from non-AM mesocosms (i.e., those that had not been inoculated with *R. irregularis* spores at the beginning of the experiment) were not colonized by AM fungi at the end of the experiment.

### Biomass harvest and isotope ratio mass spectrometry

Four months after transplanting, plant roots and aboveground tissue and fungal hyphae growing around roots were harvested and dried at 50 °C for 48 h. Fungal hyphae were harvested by floating hyphal fragments out of soil samples. Briefly, 30 g of soil were agitated vigorously in 100 mL water. After 2 min, soil was allowed to settle for 30 s and solution was decanted and filtered through 10-µm nylon filters. Large particulate matter was removed, remaining hyphal fragments were rinsed thoroughly with water, and fragments were dried as described above. Total N, C, and isotope ratios were measured using a Delta V Isotope Ratio Mass Spectrometer (Thermo Scientific, Germany) coupled to a Carlo Erba NC2500 Elemental Analyzer (Italy).

### Net N mineralization

Twenty grams of autoclaved sand, 1 g organic matter, and 0.25 g of fresh soil from the perennial switchgrass fields fertilized with 0, 28, or 196 kg N ha^−1^ per year were mixed, brought to 50% water holding capacity, and added to 60 ml glass jars. These jars were placed inside a larger glass jar with a tight-fitting lid. Eight replicates were used per treatment—four replicates were harvested for an initial time point and four replicates were incubated in the dark at 30 °C for 2 weeks. At both the initial and final time point, NO_3_^−^ and NH_4_^+^ were extracted from soil with 2 N KCl and measured (Bran and Luebbe Autoanalyzer, SPX, Charlotte, NC).

### PLFA analysis

Lipids were extracted from lyophilized organic matter samples collected from each mesocosm at the end of the experiment^39^. Briefly, the extracted lipids were fractionated into neutral lipids, glycolipids, and polar lipids on a silica acid column (Bond Elut, Varian Inc., Palo Alto, CA, USA) by successive elution with chloroform, acetone and methanol. The chloroform fraction (containing the neutral lipids) and the methanol fraction (containing the phospholipids) were subjected to mild alkaline methanolysis to transform the PLFAs and the NLFAs into free fatty acid methyl esters. These were analyzed on a gas chromatograph with a flame ionization detector and a 50 m HP5 capillary column. The PLFA 18:2ω6,9 was used as an indicator of saprotrophic fungi, the sum of the PLFAs i15:0, a15:0, 15:0, i16:0, 10Me16:0, 16:1ω7, i17:0, a17:0, cy17:0, 18:1ω7, cy19:0 was used as an indicator of bacterial biomass^40^. The PLFA 16:1ω5 is sometimes used as an indicator of AM fungal biomass, but can also be produced by other microbes^41^. Unlike all other PLFAs measured here, the PLFA 16:1ω5 was depleted in ^13^C. This is a strong indication that this PLFA represents the lipid biomass of AM fungi, which derive their C from plant photosynthates that are depleted in ^13^C, rather than the lipid biomass of other microbes, which likely would have acquired a larger proportion of their C from the ^13^C-enriched organic matter in the mesocosms.

### NanoSIMS analysis

Microbes growing in the organic matter were collected from the mesocosms after harvest, desiccated onto Si-wafer sample holders, and sputter coated with a layer of Au/Pd (~30 nm) to avoid charging during the NanoSIMS measurements (Cameca NanoSIMS 50 L, Lehrstuhl für Bodenkunde, TU München, Germany)^42^. The Cs^+^ primary ion beam was used with a primary ion impact energy of 16 keV. Prior to final analysis, any contaminants and the Au/Pd coating layer were sputtered away at 50×50 µm using a high primary beam current (pre-sputtering). During this pre-sputtering, the reactive Cs^+^ ions were implanted into the sample to enhance the secondary ion yields. The primary beam (ca. 1.2 pA) was focused at a lateral resolution of ca. 100 nm and was scanned over the sample, with ^12^C^−^, ^13^C^−^, ^12^C^14^N^−^, and ^12^C^15^N^−^ secondary ions collected on electron multipliers with an electronic dead time fixed at 44 ns. The estimated depth resolution with 16 keV Cs^+^ ions is assumed to be approx. 10 nm. All measurements were collected in imaging mode. For ion images with a 30 × 30 µm field of view, 100 planes with a dwell time of 1 ms/pixel and 256×256 pixels were recorded. NanoSIMS images were analyzed using the Open MIMS Image plugin available within ImageJ (https://imagej.nih.gov/ij). Images were corrected for the electron multiplier dead time (44 ns), drift corrected, and summed. The ^13^C/^12^C and ^12^C^15^N/^12^C^14^N ratios were extracted from all images.

### Statistics and reproducibility

All statistical analyses were performed using the statistical computing language and environment R^43^. Most means comparisons were conducted using a Tukey’s HSD test in the lsmeans package. The Tukey’s HSD test compared the means for all treatments while averaging for the effect of blocking. First, a linear model was created including a fixed effect for treatment and a block effect to account for the spatially distributed randomized block design. Q-Q plots and plots of the residual versus fitted values were used to determine whether data met the assumptions of normality. If the raw data did not meet the assumptions of normality, a log transformation was applied and statistical analyses were conducted using the log-transformed data. The comparison of expected versus observed plant acquisition of N from organic matter was conducted using an unpaired t-test (Fig. 1b). The standard error of the expected value was calculated by taking the square root of the sum of the mean squared standard deviations measured from the plants grown alone, with soil microbes from unfertilized fields only, and with AM fungi only. The NMDS analysis of microbial PLFA profiles was conducted using the vegan package, which applies a Wisconsin double standardization. This first standardizes the PLFA quantity by maxima and then standardizes each sample by the total PLFA quantity per sample.

In order to estimate the potential contribution of the tripartite synergy between plants, AM fungi, and soil microbial communities to global annual plant N acquisition, the proportion of plant N acquired from organic matter when AM plants were grown with microbes from unfertilized fields was multiplied by (a) the proportion of acquired N that could be attributed specifically to tripartite interactions between plants, AM fungi, and soil microbes, (b) the proportion of the world’s terrestrial plants that associate with AM, and (c) a conservative estimate of Tg N assimilated by plants annually:^41^ 0.18 × 0.50 × 0.80 × 1000 = 72 Tg N per year.

### Reporting summary

Further information on research design is available in the Nature Research Reporting Summary linked to this article.

## Supplementary information

Supplementary Information

Reporting Summary

## Data Availability

The data that support the findings of this study are available in Cornell University’s digital repository eCommons^44^. NanoSIMS data are available from the authors upon request.
