# Supplementary material for: Synergies between mycorrhizal fungi and soil microbial communities increase plant nitrogen acquisition
Source: Commun Biol. 2019 Jun 21;2:233. doi: 10.1038/s42003-019-0481-8 (PMC6588552; doi:10.1038/s42003-019-0481-8)
Supplement: Supplementary file 1 — Supplementary Information [file 42003_2019_481_MOESM1_ESM.docx]

**Supplementary Information**

**Synergies between mycorrhizal fungi and soil microbial communities increase plant nitrogen acquisition**

**Supplementary Figures**


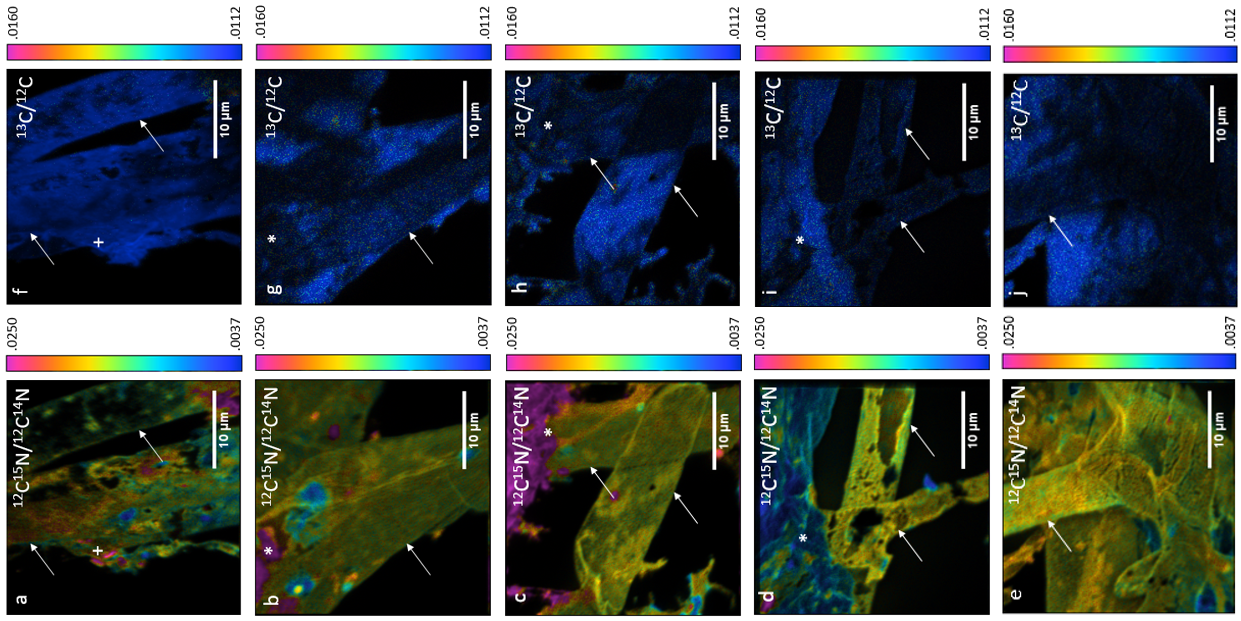


**Supplementary Figure 1. Nano-scale secondary ion mass spectrometry (NanoSIMS) images of microbes and organic matter.** The ^12^C^15^N/^12^C^14^N (a-e) and ^13^C/^12^C (f-j) isotope ratios are shown in a color scale starting with natural abundance values in dark blue (.003676 and .0111802, respectively). Fungal hyphae are indicated by arrows. Organic matter is indicated with an asterisk. Bacteria are indicated with a plus sign.

**Supplementary Tables**

**Supplementary Table 1. Plant and fungal δ ^13^C.**

| **Treatment** | **Tissue** | **δ^13^C (± SE)** |
| --- | --- | --- |
| **control** | fungal hyphae | -15.46 (0.61) a |
|  | roots | -27.63 (0.11) c |
|  | shoots | -30.20 (0.10) e |
| **+ microbes (N0)** | fungal hyphae | -14.91 (1.07) a |
|  | roots | -27.71 (0.09) c |
|  | shoots | -30.28 (0.18) e |
| **+ AM fungi** | fungal hyphae | -19.37 (0.76) b |
|  | roots | -28.67 (0.19) d |
|  | shoots | -31.35 (0.23) f |
| **+ AM fungi**  **+ microbes (N0)** | fungal hyphae | -18.36 (0.83) b |
|  | roots | -29.95 (0.30) de |
|  | shoots | -31.67 (0.25) f |

Relative ^13^C depletion is shown in δ^13^C compared to Pee Dee Belemnite. The standard error is shown in parentheses (n = 7 biologically independent samples). Letters denote the results of a Tukey’s HSD test (p < 0.05).

Both plant tissues and extraradical AM fungi were depleted in ^13^C compared to the enriched organic matter added to the mesocosms (which had a δ^13^C of 49.5 ‰), showing that both plants and AM fungi took up N from the organic matter primarily in mineral rather than in organic form. Plant tissues and fungal hyphae grown in mesocosms with AM fungi were more depleted in ^13^C than plant tissues and fungal hyphae grown without AM fungi. The magnitude of relative ^13^C depletion in tissues harvested from mesocosms with AM fungi may be a sign of increased photosynthesis under these conditions.

**Supplementary Notes**

**Alternative routes for plant uptake of organic N**

Surprisingly, even plants grown without the addition of a live soil microbial community containing decomposers acquired notable quantities of N from the organic matter (Fig. 1b and 1e). Control plants grown without any microbial inoculum and plants grown with AM fungi derived 3.3 and 7.5% of their total N from the organic matter, respectively. There are several possible routes through which plants and AM fungi could acquire N from organic matter in the absence of soil microbes with greater saprotrophic capacity. First, it is likely that some of the N in the organic matter was present in a form that was readily available for plant uptake (e.g., NH_4_^+^, NO_3_^-^, amino acids, or other small molecules). Second, despite surface sterilization of the seeds and autoclave treatment of the growing medium to minimize the background microbial community, the growth chamber environment was not completely sterile and may have supported some microbial growth and associated organic matter mineralization over the course of the experiment. Third, it is possible that the roots exuded organic acids and enzymes that contributed to soil organic matter decomposition, thereby liberating some of the N for plant and fungal acquisition^1-3^. The latter is a mechanism that is rarely considered in models of terrestrial N cycling and plant nutrition^4,5^. Our observations suggest that this may play an important role in plant N acquisition from soil organic matter and warrants further research.

**References**

1 Godlewski, M. & Adamczyk, B. The ability of plants to secrete proteases by roots. *Plant Physiol. Bioch.* **45**, 657-664 (2007).

2 Adamczyk, B., Godlewski, M., Zimny, J., & Zimny, A. Wheat (*Triticum aestivum*) seedlings secrete proteases from the roots and, after protein addition, grow well on medium without inorganic nitrogen. *Plant Biol.* **10**, 718-724 (2008).

3 Kohli, A., Narciso, J. O., Miro, B., & Raorane, M. Root proteases: reinforced links between nitrogen uptake and mobilization and drought tolerance. *Physiol. Plant.* **145**, 165-179 (2012)

4 Weil, R. R. & Brady, N. C. Nitrogen and sulfur economy of soils. In: *The Nature and Properties of Soils.* 15^th^ ed. Upper Saddle River, NJ: Pearson, 601-642 (2017).

5 Gruber, N. & Galloway, J. N. An Earth-system perspective of the global nitrogen cycle. *Nature* **451**, 293-296 (2008).
